# Long non-coding RNA *TUG1* is downregulated in Friedreich’s ataxia

**DOI:** 10.1093/braincomms/fcae170

**Published:** 2024-05-15

**Authors:** Mert Koka, Hui Li, Rumana Akther, Susan Perlman, Darice Wong, Brent L Fogel, David R Lynch, Vijayendran Chandran

**Affiliations:** Department of Pediatrics, College of Medicine, University of Florida, Gainesville, FL 32610, USA; Department of Pediatrics, College of Medicine, University of Florida, Gainesville, FL 32610, USA; Department of Pediatrics, College of Medicine, University of Florida, Gainesville, FL 32610, USA; Department of Neurology, David Geffen School of Medicine, University of California, Los Angeles, CA 90095, USA; Department of Neurology, David Geffen School of Medicine, University of California, Los Angeles, CA 90095, USA; Clinical Neurogenomics Research Center, David Geffen School of Medicine, University of California, Los Angeles, CA 90095, USA; Department of Neurology, David Geffen School of Medicine, University of California, Los Angeles, CA 90095, USA; Clinical Neurogenomics Research Center, David Geffen School of Medicine, University of California, Los Angeles, CA 90095, USA; Department of Human Genetics, David Geffen School of Medicine, University of California, Los Angeles, CA 90095, USA; Division of Neurology, Children’s Hospital of Philadelphia, Philadelphia, PA 19104, USA; Department of Pediatrics, College of Medicine, University of Florida, Gainesville, FL 32610, USA; Department of Neuroscience, College of Medicine, University of Florida, and McKnight Brain Institute, Gainesville, FL 32610, USA

**Keywords:** Friedreich's ataxia, *TUG1*, biomarker, frataxin knockdown, gene expression

## Abstract

Friedreich's ataxia is a neurodegenerative disorder caused by reduced frataxin levels. It leads to motor and sensory impairments and has a median life expectancy of around 35 years. As the most common inherited form of ataxia, Friedreich’s ataxia lacks reliable, non-invasive biomarkers, prolonging and inflating the cost of clinical trials. This study proposes *TUG1*, a long non-coding RNA, as a promising blood-based biomarker for Friedreich’s ataxia, which is known to regulate various cellular processes. In a previous study using a frataxin knockdown mouse model, we observed several hallmark Friedreich’s ataxia symptoms. Building on this, we hypothesized that a dual-source approach—comparing the data from peripheral blood samples from Friedreich’s ataxia patients with tissue samples from affected areas in Friedreich’s ataxia knockdown mice, tissues usually unattainable from patients—would effectively identify robust biomarkers. A comprehensive reanalysis was conducted on gene expression data from 183 age- and sex-matched peripheral blood samples of Friedreich’s ataxia patients, carriers and controls and 192 tissue data sets from Friedreich’s ataxia knockdown mice. Blood and tissue samples underwent RNA isolation and quantitative reverse transcription polymerase chain reaction, and frataxin knockdown was confirmed through enzyme-linked immunosorbent assays. *Tug1* RNA interaction was explored via RNA pull-down assays. Validation was performed in serum samples on an independent set of 45 controls and 45 Friedreich’s ataxia patients and in blood samples from 66 heterozygous carriers and 72 Friedreich’s ataxia patients. *Tug1* and *Slc40a1* emerged as potential blood-based biomarkers, confirmed in the Friedreich’s ataxia knockdown mouse model (one-way ANOVA, *P* ≤ 0.05). *Tug1* was consistently downregulated after *Fxn* knockdown and correlated strongly with *Fxn* levels (*R*^2^ = 0.71 during depletion, *R*^2^ = 0.74 during rescue). *Slc40a1* showed a similar but tissue-specific pattern. Further validation of *Tug1*'s downstream targets strengthened its biomarker candidacy. In additional human samples, *TUG1* levels were significantly downregulated in both whole blood and serum of Friedreich’s ataxia patients compared with controls (Wilcoxon signed-rank test, *P* < 0.05). Regression analyses revealed a negative correlation between *TUG1* fold-change and disease onset (*P* < 0.0037) and positive correlations with disease duration and functional disability stage score (*P* < 0.04). This suggests that elevated *TUG1* levels correlate with earlier onset and more severe cases. This study identifies *TUG1* as a potential blood-based biomarker for Friedreich’s ataxia, showing consistent expression variance in human and mouse tissues related to disease severity and key Friedreich’s ataxia pathways. It correlates with frataxin levels, indicating its promise as an early, non-invasive marker. *TUG1* holds potential for Friedreich’s ataxia monitoring and therapeutic development, meriting additional research.

## Teaser

Koka *et al*. report that the long non-coding RNA *TUG1* is downregulated in Friedreich's ataxia and closely correlates with disease severity and frataxin levels. This suggests *TUG1*'s utility as an early, non-invasive blood-based biomarker for Friedreich's ataxia, with potential implications for disease monitoring and therapeutic development.

## Graphical Abstract

**Graphical Abstract:**
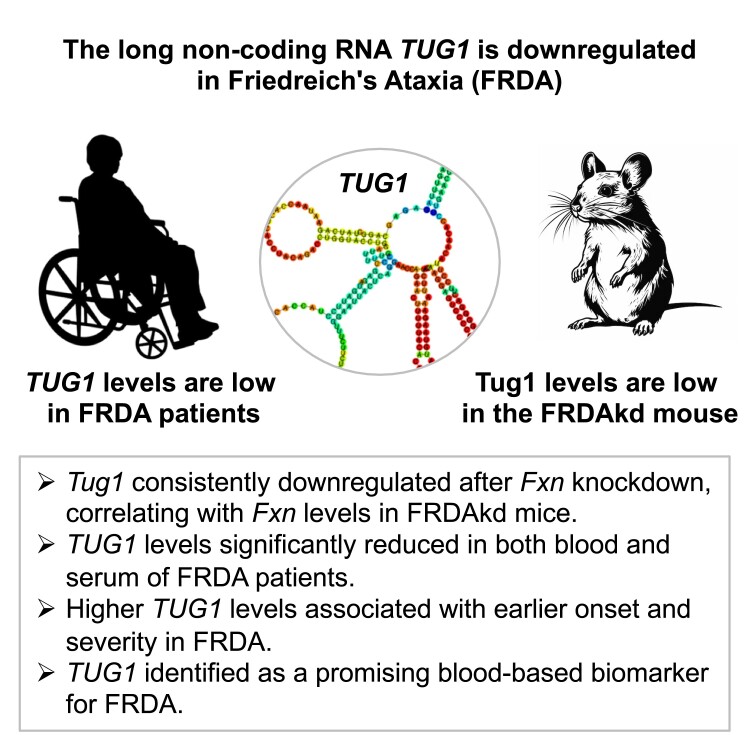

## Introduction

Friedreich's ataxia is a debilitating neurodegenerative disorder characterized by the progressive degeneration of the nervous system, resulting in significant motor and sensory impairments.^1,2^ The disease ultimately leads to severe physical disability and reduced life expectancy, with the median age of death being 35 years.^3,4^ Friedreich's ataxia, being the most common inherited ataxia, is caused by a guanine–adenine–adenine (GAA) trinucleotide repeat expansion in the first intron of the frataxin (FXN) gene.^1,2^ This genetic change has a strong correlation with disease severity and its onset.^5^ Notably, while heterozygous carriers of the GAA expansion remain asymptomatic, their prevalence ranges from 1:60 to 1:110 in European populations.^6,7^ The need for an effective treatment for Friedreich's ataxia is challenged by the extensive and expensive clinical trials necessary for drug validation.^8^ Therefore, there is an increased emphasis on identifying molecular biomarkers that can quickly monitor disease progression. These biomarkers may expedite evaluations of possible treatments, enhancing patient care and prognosis.^8^

Recent advancements in Friedreich's ataxia research have enabled the assessment of over 20 potential therapeutic interventions in clinical trials.^8^ The Food and Drug Administration (FDA) has recently approved omaveloxolone, the first drug for the treatment of Friedreich's ataxia.^9^ However, its moderate efficacy and associated side effects underscore the continuing need for more effective treatments.^9^ A major obstacle in developing these therapies is the time-consuming and costly nature of clinical trials.^8^ This highlights the urgent need for reliable molecular biomarkers for quicker, evidence-based evaluation of potential treatments. Frataxin, central to Friedreich's ataxia pathology, exists in two primary forms: mitochondrial frataxin (frataxin-M) and an erythrocyte-specific variant (frataxin-E). The full-length precursor, a 210-amino acid protein, undergoes mitochondrial processing to yield frataxin-M, while frataxin-E, lacking a mitochondrial targeting sequence, is found predominantly in erythrocytes.^10-12^ This distribution challenges the direct measurement of frataxin in serum or plasma, as it is not commonly secreted into systemic circulation. Moreover, the presence of these proteoforms in different cell types adds variability to frataxin measurements in whole blood.^10-12^ Given the challenges in detecting FXN directly, and the intricacies of frataxin's distribution, identifying alternative biomarkers that correlate with FXN levels or disease progression is paramount. For Friedreich's ataxia therapies targeting increased frataxin levels, monitoring frataxin itself is the most direct approach. However, for symptomatic treatments of Friedreich's ataxia, additional biomarkers measurable in peripheral tissues, such as blood samples, are essential. Discovering such biomarkers in Friedreich's ataxia has been challenging, primarily because of difficulties accessing affected tissues at various disease stages and the lack of animal models that faithfully mimic human disease manifestations.

In our prior research, we presented the Friedreich's ataxia knockdown (FRDAkd) mouse model, which closely replicates the symptoms seen in Friedreich's ataxia patients, offering insights into disease progression and the potential for recovery when frataxin expression is restored.^13^ In this model, where FXN expression is reduced, key Friedreich's ataxia symptoms are evident, including ataxia, early mortality, muscle atrophy, degeneration of the dorsal root ganglia (DRG), impaired structural integrity of spinal cord axons and myelin, cardiomyopathy and iron overload.^13^ Utilizing this model, this study aims to address the absence of a high confidence molecular biomarker for Friedreich's ataxia. We hypothesize that through the integrative analysis of genomic data sets from (i) Friedreich's ataxia patient samples and (ii) samples from the tissues of FRDAkd mice, which are primarily affected in Friedreich's ataxia patients, we can identify robust biomarkers intricately associated with the severity and progression of the disease.

In this study, we focus on the long non-coding RNA (lncRNA) taurine-upregulated gene 1 (*TUG1*) as a prospective biomarker for Friedreich's ataxia. By analysing genomics data from both human and mouse models, we detected reduced expression of *TUG1* in Friedreich's ataxia. Our results reveal a significant correlation between *Tug1* and *Fxn* levels, highlighting the association of *Tug1* downregulation with the disease. We suggest that evaluating *TUG1* levels could provide a non-invasive metric for disease onset, progression and severity. Such a biomarker could significantly enrich the therapeutic landscape of Friedreich's ataxia, enabling prompt therapeutic interventions and improving long-term evaluations. In this paper, we report these findings and discuss their implications for the prognosis and management of Friedreich's ataxia. These observations, based on rigorous genomics analysis and a deep understanding of Friedreich's ataxia biology, underscore the potential of lncRNA *TUG1* as a promising molecular biomarker in Friedreich's ataxia.

## Materials and methods

### Analysis of human and mouse gene expression data

**Figure 1:**
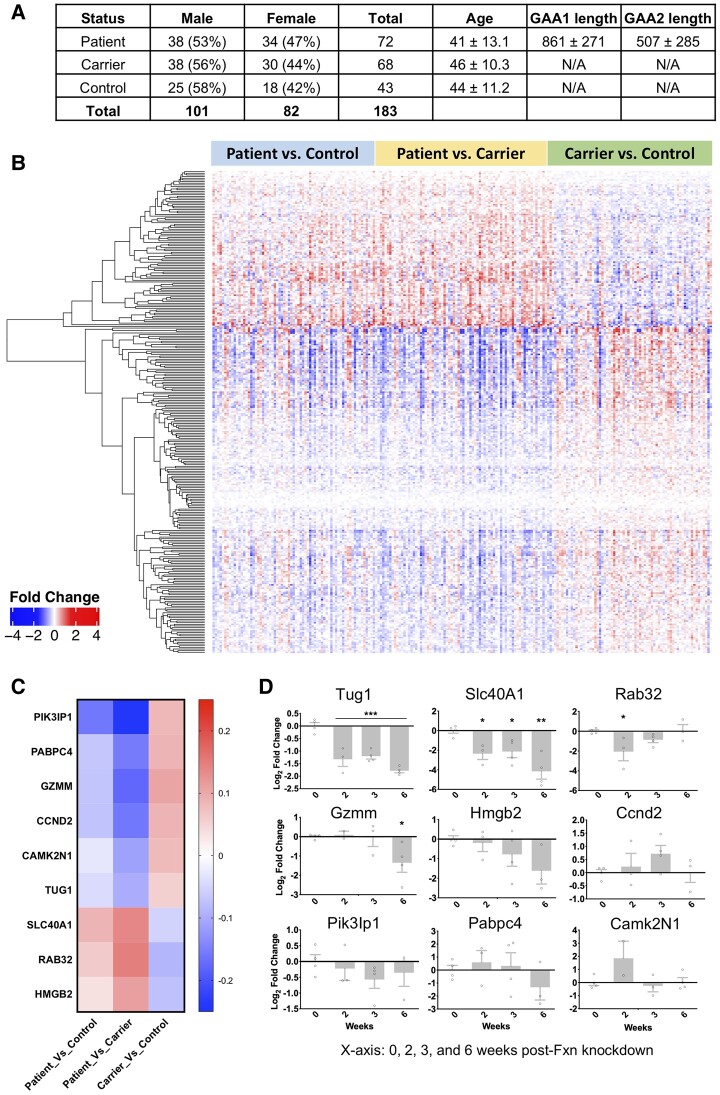
**Differential gene expression in whole blood of Friedreich's ataxia patients.** (**A**) Demographic breakdown of the participants, featuring sex- and age-matched samples used for the analysis. GAA1 and GAA2 lengths represent the number of GAA triplet repeat expansions in the first and second alleles, respectively, of the FXN gene, which are characteristic of Friedreich's ataxia. (**B**) A heat map representing significant gene up- and downregulation (depicted in rows) in whole blood from Friedreich's ataxia patients (*n* = 72), carriers (*n* = 68) and controls (*n* = 43), sourced from GEO data set GSE102008. In the heatmap, intensities of red (indicating gene upregulation) and blue (indicating gene downregulation) correspond to the respective levels of gene expression changes. (**C**) A heat map showing the top nine differentially expressed genes in Friedreich's ataxia patient's whole blood, selected for subsequent validation. (**D**) Preliminary screening of expression levels of candidate genes in the whole blood of FRDAkd mice, normalized to Hprt1. Expression levels were measured post-treatment with dox (Fxn knockdown) from Week 0 to Week 6 using RT-qPCR analyses. The individual circles represent data points from each animal, corresponding to the expression level of each candidate gene at 0, 2, 3 and 6 weeks post-*Fxn* knockdown. The up- and downregulation of these genes, as a result of dox-induced *Fxn* knockdown, are denoted by positive and negative signals, respectively. Sample size ranges from *N* = 3 to *N* = 5. Statistical analyses were performed using a one-way ANOVA and Welch's *t*-test. Data are presented as mean ± SEM. Significance is indicated as follows: *P ≤ 0.05, **P ≤ 0.01 and ***P ≤ 0.001.

A total of 733 peripheral blood samples from Friedreich's ataxia patients (411), carriers (228) and controls (94) were reanalysed to identify blood-based biomarker for Friedreich's ataxia [Gene Expression Omnibus (GEO) data set: GSE102008].^14^ To avoid any confounding effect in this data series, we conducted differential gene expression analyses on age- and sex-matched samples (Fig. 1A). We established a subcohort of 183 samples from the GEO data set, with stringent matching for age and sex. The subcohort included 72 Friedreich's ataxia patients (53% male, 47% female, average age 41), 68 carriers (56% male, 44% female, average age 46) and 43 controls (58% male, 42% female, average age 44). This careful selection resulted in a subcohort that mirrors the larger data set with a gender balance of 101 males and 82 females and relevant age averages across the groups, permitting accurate differential gene expression analyses. For the mouse data, a total of 192 microarray data sets from 64 RNA samples derived from FRDAkd mice were analysed to compare and examine the overlap of the differentially expressed genes obtained from Friedreich's ataxia patient data and mouse data (GEO data set: GSE98790).^13^ Both raw data were log transformed and checked for outliers. Interarray Pearson correlation and clustering based on variance were used as quality control measures. Quantile normalization was used and contrast analysis of differential expression was performed by using the LIMMA package (RRID:SCR_010943). Briefly, a linear model was fitted across the data set, contrasts of interest were extracted and differentially expressed genes for each contrast were selected using an empirical Bayes test statistic.^15^

### Animal study design and ethics

All animal experiments were carried out in accordance with relevant guidelines and regulations and upon the approval of an Institutional Animal Care and Use Committee (IACUC) at the University of Florida (protocol number # 201909663) and in compliance with the ARRIVE guidelines. Wild-type mice were C57BL/6J from the Jackson Laboratory, and transgenic mice were FRDAkd mice in C57BL/6J background. Mice were sorted into four different groups: wild type with doxycycline (Wt + dox), transgenic with doxycycline (Tg + dox), without doxycycline (Tg − dox) and transgenic with dox removal–rescue (Tg + res). The number of mice (*N*) was four for each time point. The male to female ratio was 1:1 for all groups. Animals were to be euthanized at Weeks 0, 2, 3, 4, 6, 8, R1, R2, R3, R4, R6 and R8 (R = rescue − dox removal). Transgenic with dox and wild type with dox animals were given 2000 mg/kg dox hyclate (∼87% dox) diet, TD.09633 from Envigo. Transgenic without dox animals were given normal diet. Transgenic with rescue were switched from dox hyclate diet to normal diet.

### Mice tissue and blood sample collection protocol

Animals were deeply anaesthetized with isoflurane, and cardiac puncture was performed to collect the blood in ethylenediamine tetraacetic acid (EDTA)-covered RNAse-free tube. The blood was then centrifuged at 3000 × g for 15 min. The supernatant (the plasma) was collected and snap frozen with liquid nitrogen. Mice were placed on petri dish on ice, and dissection of the heart, liver, brain and muscle from the femur was performed. The spinal column was isolated, and hydraulic extrusion of the spinal cord was performed with ice-cold 1× phosphate-buffered saline (PBS). All tissues were cut and rinsed in ice-cold 1× PBS and quickly transferred to 1.7 mL sterile, RNAse-free tubes to be snap frozen with liquid nitrogen. All samples were stored in −80°C.

### RNA isolation, cDNA preparation and quantitative reverse transcription polymerase chain reaction protocols

Tissue samples of the blood, muscle, heart, spinal cord, brain and liver from FRDAkd mice were retrieved from a −80°C freezer for RNA isolation. The PAXgene blood RNA kit (cat #762164) was used for blood samples, while the miRNeasy mini kit (cat #2170040) was utilized for other tissues. SuperScript VILO master mix was employed to synthesize cDNA from the extracted RNA. Depending on the tissue type, total RNA concentrations ranging from 0.5 to 1.0 µg were used to prepare 10 µL cDNA aliquots as per the manufacturer's guidelines. For quantitative reverse transcription polymerase chain reaction (qRT-PCR), iTaq Universal SYBR Green Supermix was used. Each well contained 0.2 µL of the cDNA prep and 500 nM each of forward and reverse primers. The qRT-PCR was conducted using the Bio-Rad CFX96 real-time PCR system. Melt curve analysis was performed to confirm the presence of a single amplicon for each of the primer sets (Table 1).

**Table 1:** List of primers

#	Primer name	Primer sequence	#	Primer name	Primer sequence
1	Hprt1_forward	GCTCGAGATGTCATGAAGGA	26	MMP2_reverse	TGCATTGCCACCCATGGTAAA
2	Hprt1_reverse	AACTTTTATGTCCCCCGTTG	27	CASP1_forward	CGTACACGTCTTGCCCTCAT
3	RAB32_forward	GCTCTTCTCCCAGCACTACC	28	CASP1_reverse	AACTTGAGCTCCAACCCTCG
4	RAB32_reverse	GTCCTGCTGTCCCAGTTGAG	29	LCP1_forward	ACTGAGAATTCAAGTCTGTCACC
5	PABPC4_forward	GGCTTCCAAGGAATGCCAAG	30	LCP1_reverse	AGCTGATGTATCCGTTGCCA
6	PABPC4_reverse	CCGGAGCATTACCAGTTGGA	31	ACADS_forward	TAGCCATGCAAACCCTGGAC
7	HMGB2_forward	AGGGCAAAAGTGAAGCAGGA	32	ACADS_reverse	TTGCGGTTCTCGGCATACTT
8	HMGB2_reverse	CCTCCTCATCTTCTGGTTCGT	33	BDNF_forward	TCTTTTCCGAGGTTCGGCTC
9	CAMK2N1_forward	TACGGCGACGAGAAGCTGAG	34	BDNF_reverse	CAGCCTACACCGCTAGGAAG
10	CAMK2N1_reverse	GAAGAAGTTGTTGGTGTCCTGC	35	ACSL4_forward	GAAGAAATCTAAAAACGCTATGGCA
11	CCND2_forward	TTCAAGTGCGTGCAGAAGGA	36	ACSL4_reverse	TCCAGAGTATCTGCTCCAGGG
12	CCND2_reverse	GCCAAGAAACGGTCCAGGTA	37	CD86_forward	CTGTATAAGGACGCCCAGGAG
13	TUG1_forward	CAGCCTACAGACCTGGTACTTG	38	CD86_reverse	ACAGCAGCATTCCCGAAGAT
14	TUG1_reverse	TGGTCCACTGGAATGGTGTC	39	LY9_forward	GGCTCTAGGTCCACTCTCTGA
15	SLC40A1_forward	GTGACACAGTTGCTGCAAGA	40	LY9_reverse	TCACTGTTGGAGGTGTTTCCT
16	SLC40A1_reverse	AGGATTTGGGGCCAAGATGA	41	GTDC1_forward	ACAGGGAAGGGGACTACAGG
17	GZMM_forward	GTAACAACAGCCGCTTCTGG	42	GTDC1_reverse	ATCCAATCTTGTACCGGCTCG
18	GZMM_reverse	CCTCCAGAGTCACCCTTGC	43	OMG_forward	TTTCTCACGCCTGGCATCTT
19	PIK3IP1_forward	GCCTATGGATCTGGAGGCTG	44	OMG_reverse	CCTGAACAGTCCACATGCCT
20	PIK3IP1_reverse	GGCCAACCAGTTGAGGCAG	45	CLCN3_forward	ACTCAAAGTCTTTCTTAGCACAGT
21	VSIG4_forward	TCATTGAGCTCCGTGTTCGG	46	CLCN3_reverse	TCAAACAACAGCTGGGAGACA
22	VSIG4_reverse	GGAGTGCAGGGTTGTAGGTG	47	APBB1IP_forward	CTCCTCCCAGCAGTCAAGAT
23	NEDD1_forward	TGGGCAGCGTTTCGGATAAT	48	APBB1IP_reverse	TAAACTCTTCTCGGGGTGGG
24	NEDD1_reverse	AAGCTGGGGCTTTGTGTGTA	49	CRYM_forward	ATGCGCTCACCACCAAGTTA
25	MMP2_forward	CATGGCCTCACACACGGTA	50	CRYM_reverse	GAGAAGCACCGATGCCTGAT

### Validation of frataxin knockdown using enzyme-linked immunosorbent assays in heart tissue

To validate the knockdown of frataxin, heart tissue samples were selected for enzyme-linked immunosorbent assay (ELISA) analysis. Snap-frozen heart tissue aliquots from all 40 mice were retrieved from a −80°C storage and weighed in new tubes. For protein extraction, 10 µL of 1× ELISA lysis buffer (Abcam ab176112 ELISA kit) per milligram of tissue was used in conjunction with a pestle tissue homogenizer. Protein concentration was quantified using the BCA assay kit (cat #23225). Subsequently, 30 µg of total protein from each heart sample was loaded into ELISA wells, and the protocol outlined in the ELISA kit was followed. Frataxin concentrations in the samples were interpolated using a second-order polynomial regression model based on a standard curve generated from a human lyophilized recombinant frataxin protein provided in the kit. Data were plotted using Prism 6.

### RNA pull-down assay to investigate *Tug1* RNA interactome

To explore the RNA interactome of *Tug1*, a previously established RNA pull-down protocol for lncRNAs was employed.^16^ The secondary structure of lncRNA *Tug1* was predicted using RNAstructure Webserver, and an antisense biotinylated DNA oligonucleotide probe with minimal probability of internal base pairing was designed via the Fold algorithm.^17^ A non-specific DNA oligonucleotide probe served as a negative control. Heart tissues were initially crosslinked with paraformaldehyde, lysed and sonicated. Lysates were mixed with a hybridization buffer, and an input sample was frozen until use. Subsequent hybridization involved adding biotinylated DNA probes to the lysates for 4 h, followed by overnight incubation with streptavidin beads. The beads were magnetically separated and washed several times. Proteinase K treatment was performed before RNA isolation using the miRNeasy mini kit (cat #2170040). Reverse transcription was carried out with SuperScript IV VILO master mix with ezDNase (cat #11766050). The assay was analysed using reverse transcription quantitative PCR (RT-qPCR) to determine relative enrichment of target molecules in comparison with input samples.

### Clinical sample acquisition and ethical approvals

Acquisition protocols for human clinical samples were approved by the Institutional Review Boards (IRBs) at the University of California, Los Angeles (UCLA) and the Children's Hospital of Philadelphia (CHOP). Informed written consent was obtained from all participants or from legal guardians in the case of subjects under 18 years. The sample set comprised 45 healthy controls and 45 Friedreich's ataxia patients for serum samples. Additionally, blood samples were collected from 66 heterozygous carriers of the *FXN* gene with GAA repeats (unaffected) and 72 Friedreich's ataxia patients.

### Venous blood and serum sample collection and storage

Blood samples were collected from each participant using 8.5 mL purple-cap Vacutainer EDTA tubes (Becton, Dickinson and Company, NJ, USA, cat #367861). The tubes were gently inverted 8–10 times postcollection for anticoagulant mixing. The samples were transferred into 2.4 mL Eppendorf tubes (Eppendorf AG, Hamburg, Germany) with sterile pipettes and immediately stored at −80°C in an ultralow freezer. For serum, blood samples were allowed to clot at room temperature for 30 min before undergoing centrifugation at 1500 × g for 10 min at 4°C using a refrigerated centrifuge (Eppendorf AG, Hamburg, Germany). The supernatant serum was pipetted into sterile 1.5 mL Eppendorf tubes and stored at −80°C until further analysis.

### Statistical analysis and data interpretation

Data normality was assessed using Kolmogorov–Smirnov, Shapiro–Wilk, or D'Agostino–Pearson tests based on the data set size. For normally distributed data, a one-way or two-way ANOVA was employed for statistical analysis. In cases where the data did not conform to a normal distribution, alternative statistical tests were applied as indicated in the respective figure legends. Grouped data were analysed using a one- or two-way ANOVA with appropriate *post hoc* multiple comparison tests. All statistical analyses were conducted using the GraphPad Prism 9 software (GraphPad Prism, RRID: SCR_002798). A *P*-value of ≤0.05 was considered statistically significant. Results were calculated based on *N* = 4 or more per time point and are presented as mean ± SEM.

## Results

### Blood-based biomarker discovery in Friedreich's ataxia

In this study, we reanalysed an extensive series of previously published unbiased gene expression profiles comprising 733 individuals, including 411 Friedreich's ataxia patients, 228 carriers and 94 controls, with the aim of identifying blood-based biomarkers specific to Friedreich's ataxia.^14^ Differential gene expression analyses were conducted on age- and sex-matched 183 samples, made up of 72 patients, 68 carriers and 43 controls, to eliminate any confounding effects in these data series (Fig. 1A). This analysis identified 293 genes that were differentially expressed with a false discovery rate (FDR) of <10% (Fig. 1B). Subsequent functional annotation analysis of these transcripts revealed predominant Gene Ontology categories, such as adaptive and innate immune response. This finding aligns with previous research, where immune system activation was one of the earliest regulated pathways post-*Fxn* knockdown.^13,14^ Thus, utilizing the differentially expressed 293-gene list derived from Friedreich's ataxia patient whole blood data, we postulate that it is feasible to rank and validate these genes for the identification of potential biomarkers for Friedreich's ataxia.

### Biomarker validation of *Tug1* and *Slc40a1* in FRDAkd mice

Utilizing gene expression data from the FRDAkd mouse model,^11^ we examined the overlap of the 293 differentially expressed genes obtained from Friedreich's ataxia patient data and mouse data. This analysis focused on three tissues primarily affected in Friedreich's ataxia: the heart, DRG neurons and the cerebellum. We investigated these tissues after *Fxn* knockdown, achieved through dox treatment, and subsequent rescue following dox removal.^11^ We found 49 genes that were differentially expressed in both Friedreich's ataxia patient data and FRDAkd mouse gene expression data, with an FDR of <5%. These genes were then ranked based on consistency across samples and a non-parametric Kruskal–Wallis *P* < 1.2 × 10^−4^, and the top nine genes (*Slc40A1*, *Rab32*, *Tug1*, *Pabpc4*, *Gzmm*, *Hmgb2*, *Camk2N1*, *Ccnd2* and *Pik3Ip1*) were selected for further validation (Fig. 1C). To validate these nine genes and determine if these candidate biomarkers are direct targets due to *Fxn* knockdown, we performed a focused examination. Specifically, we assessed whether they were differentially expressed in the whole blood as early as 2 weeks after *Fxn* knockdown in FRDAkd mice. This process aimed to discern if the observed alterations in gene expression were a direct consequence of changes in *Fxn* levels, thus strengthening the hypothesis that these genes could serve as potential biomarkers for Friedreich's ataxia. Their expression levels were assessed by qRT-PCR at various intervals (0, 2, 3 and 6 weeks) post dox treatment. Among the nine genes, taurine-upregulated gene 1 (*Tug1*) and ferroportin-1 (*Slc40a1*) were found to be significantly differentially expressed (one-way ANOVA, *P* ≤ 0.05) as early as 2 weeks after *Fxn* knockdown in whole blood (Fig. 1D). The differential expression of *Tug1* and *Slc40a1* in intracardiac blood positions these genes as exemplary candidate biomarkers for Friedreich's ataxia, particularly appealing due to the minimal invasiveness required for patient sample collection.

### Assessment of frataxin levels and correlation with candidate biomarkers in FRDAkd mouse model

**Figure 2:**
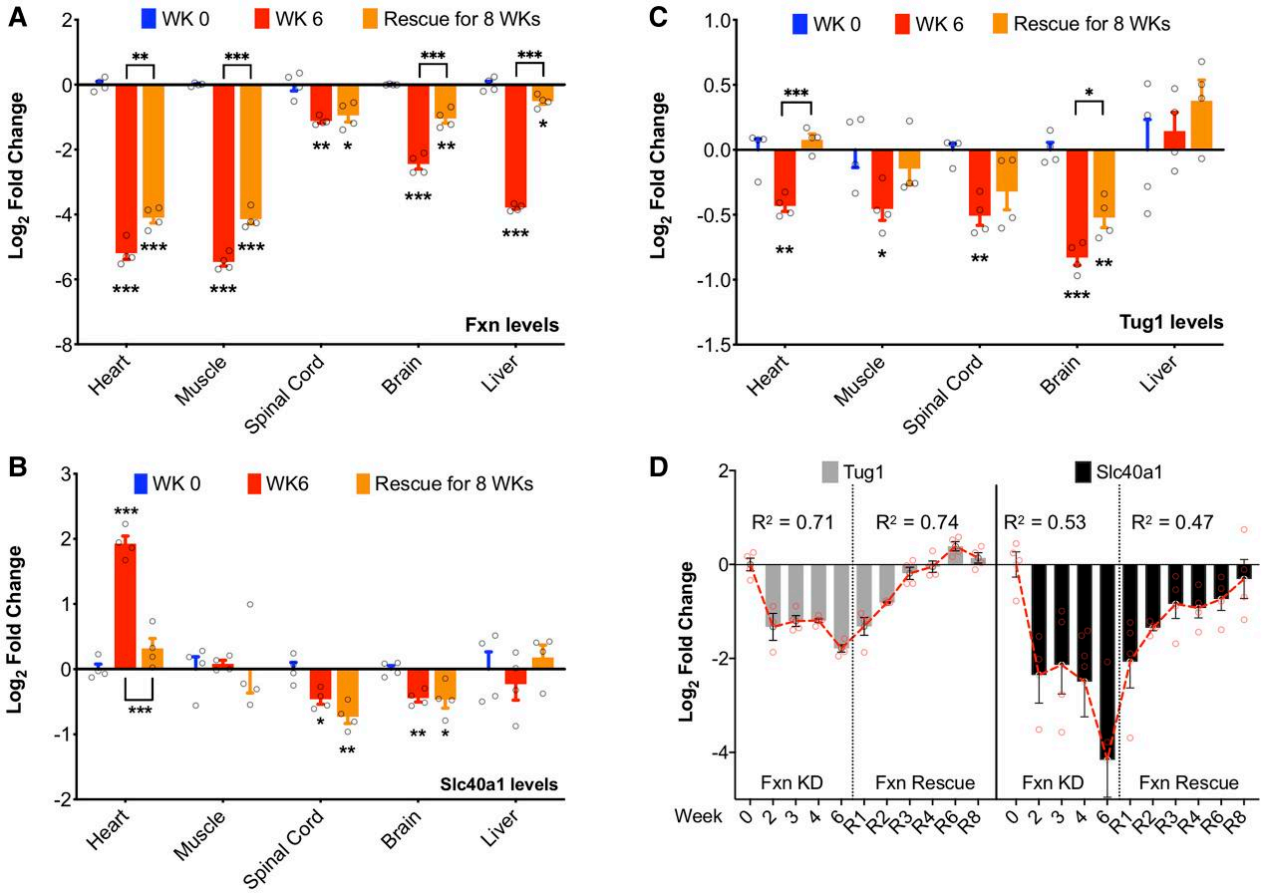
**Tissue-specific expression levels of *Fxn*, *Tug1* and *Slc40a1* in FRDAkd mice.** (**A**) *Fxn*, (**B**) *Slc40a1* and (**C**) *Tug1*, expression levels in heart, muscle, spinal cord, brain and liver tissues from FRDAkd mice treated with dox for 0 and 6 weeks (WK, *Fxn* knockdown), followed by an 8-week rescue period (*Fxn* recovery). Each time point included four samples (*n* = 4). (**D**) Linear regression models were employed to calculate *R*^2^ values based on the log_2_ fold-change of *Tug1* and *Slc40a1* expression levels, normalized to *Hprt1*, in the blood of FRDAkd mice during the *Fxn* knockdown and rescue phases. *N* = 3–5. Welch's *t*-test was used for statistical analysis. Data are presented as mean ± SEM, with significance marked as *P ≤ 0.05, **P ≤ 0.01 and ***P ≤ 0.001.

Carriers and Friedreich's ataxia patients exhibit reduced frataxin levels when compared with controls. The lateral flow immunoassays conducted on buccal cells revealed frataxin protein levels at 50.5% in carriers and 21.1% in Friedreich's ataxia patients.^18^ The FRDAkd mouse model facilitates an exploration of various frataxin-level profiles across different tissues, mirroring those observed in controls, carriers and Friedreich's ataxia patients. These profiles vary based on the dose, duration and rescue of dox treatment. This model afforded the opportunity to investigate the correlation between the expressions of the top two candidate biomarkers (*Tug1* and *Slc40a1*) at different time points and the varying levels of FXN. Utilizing ELISA on heart samples, we demonstrated the FXN-level profiles within the FRDAkd mouse model. After dox treatment, the FXN levels in the heart samples of FRDAkd mice were observed to decrease by 47% by Week 2, and 93% by Week 6 (Supplementary Fig. 1A). Extended exposure to dox treatment yielded lower FXN levels in the heart, while Tg − dox and Wt + dox remained unchanged. At the mRNA level, a significant knockdown of *Fxn* was detected at Week 6 in the heart, muscle, spinal cord, brain and liver, with evidence of partial rescue of *Fxn* expression by 8 weeks postdox removal (R8) in the heart, muscle, brain and liver (Fig. 2A).

We next delved into the examination of *Tug1* and *Slc40a1* gene expression across various tissues in the FRDAkd mouse model to explore the consistency of expression changes across different tissues. Specifically, we analysed the gene expression levels of *Tug1* and *Slc40a1* in five diverse tissues (heart, muscle, spinal cord, brain and liver) at three time points: immediately after *Fxn* knockdown at 0 and 6 weeks postdox treatment, followed by rescue at 8 weeks postdox removal (Fig. 2B and C). After reducing *Fxn* levels, we noted a marked decrease in *Slc40a1* expression in both spinal cord and brain tissues. Notably, *Slc40a1* mRNA levels did not return to normal immediately upon *Fxn* restoration, suggesting a delayed recovery process. Conversely, in the heart tissue, *Fxn* knockdown led to an upregulation of *Slc40a1*, with subsequent *Fxn* rescue resulting in a reversal of this expression pattern. In the liver and muscle tissues, there were no evident alterations in *Slc40a1* expression attributed to *Fxn* knockdown (Fig. 2B). It is essential to note that ferroportin-1 is a critical transmembrane protein involved in iron export.^19^ Previous findings have highlighted iron metabolism dysregulation in heart autopsies of Friedreich's ataxia patients.^20,21^ Moreover, iron concentrations in plasma samples were found to be significantly lower in Friedreich's ataxia patients compared with healthy controls.^22^ These observations align cohesively with our results, further emphasizing the potential role of *Slc40a1* in the pathological mechanisms underlying Friedreich's ataxia.

### Analysis of the potential of *Tug1* as a specific biomarker for Friedreich's ataxia

Our next candidate, lncRNA *Tug1*, is known to interact with the polycomb repressor complex and functions in the epigenetic regulation of transcription. *Tug1* has been shown as a regulatory factor, involved in cellular processes such as cell proliferation,^23-25^ apoptosis,^26-30^ cell cycle^24,26,31,32^ and mitochondrial bioenergetics.^33^ Notably, these processes are also primarily impacted in Friedreich's ataxia. Through our analysis, we discovered that *Tug1* was markedly downregulated in all examined tissues following *Fxn* knockdown, with the exception of the liver. During the *Fxn* restoration phase, the expression level of *Tug1* exhibited complete recovery in the heart and muscle tissues. However, there was only a limited rescue of *Tug1* expression in the spinal cord and brain, as depicted in Fig. 2C. This limited rescue can be attributed to residual levels of dox remaining in the CNS after its withdrawal. In summary, these experiments reveal a consistent expression pattern of *Tug1* in response to *Fxn* knockdown and restoration, highlighting its significant downregulation across various tissues and its potential role as a specific biomarker for Friedreich's ataxia.

### Analysis of *Tug1* and *Slc40a1* expression in whole blood during *Fxn* knockdown and rescue in FRDAkd mice

Following the preliminary screening of candidate genes at early time points, we evaluated the expression of *Tug1* and *Slc40a1* genes in whole blood across 11 specific time points, consisting of five stages during *Fxn* knockdown and six stages during *Fxn* rescue. During the *Fxn* depletion phase, both *Tug1* (*R*^2^ = 0.71) and *Slc40a1* (*R*^2^ = 0.53) exhibited a strong linear decline. Conversely, during the *Fxn* rescue phase, *Tug1* (*R*^2^ = 0.74) and *Slc40a1* (*R*^2^ = 0.47) demonstrated a marked linear increase (Fig. 2D). To control for the potential confounding effects of dox treatment, we examined the expression of *Tug1* and *Slc40a1* in wild-type mice across 0, 6 and R8 weeks of dox treatment and subsequent removal (Supplementary Fig. 1B). No significant alterations in mRNA expression of *Tug1* and *Slc40a1* were observed in wild-type animals during either dox treatment or withdrawal, supporting the notion that the changes in transgenic mice were attributable to the changes in *Fxn* levels. In the context of these findings, *Tug1* emerged as a more suitable biomarker for Friedreich's ataxia compared with *Slc40a1*. This is based on *Tug1*'s consistent expression across various tissues and patient samples, making it a reliable biomarker for Friedreich's ataxia. While *Slc40a1*'s role in iron metabolism is crucial for FRDA, *Tug1*'s robust and consistent expression profile highlights its potential as a more effective peripheral biomarker.

Our previous gene expression data in FRDAkd mice revealed significant downregulation of *Tug1* in the cerebellum and DRG throughout disease progression, paralleling the trend in *Fxn* expression (Supplementary Fig. 2A). In Friedreich's ataxia, significant neuronal loss in the dentate nuclei and extensive cerebellar damage have been reported.^4,34,35^ Interestingly, we observed that *TUG1* expression is highest in the cerebellum among all human brain regions (Supplementary Fig. 2B). Furthermore, in FRDAkd mice treated with dox (*Fxn* knockdown), we observed a downregulation of *Tug1* as early as the third week of treatment (Supplementary Fig. 2C). In summary, *Tug1*'s strong linear correlation with *Fxn* levels during knockdown and rescue phases, consistency in expression across various tissues and early detection of downregulation in specific regions such as the cerebellum present it as a suitable and compelling candidate biomarker for Friedreich's ataxia.

### *Tug1* downstream targets are altered in FRDAkd mice

**Figure 3:**
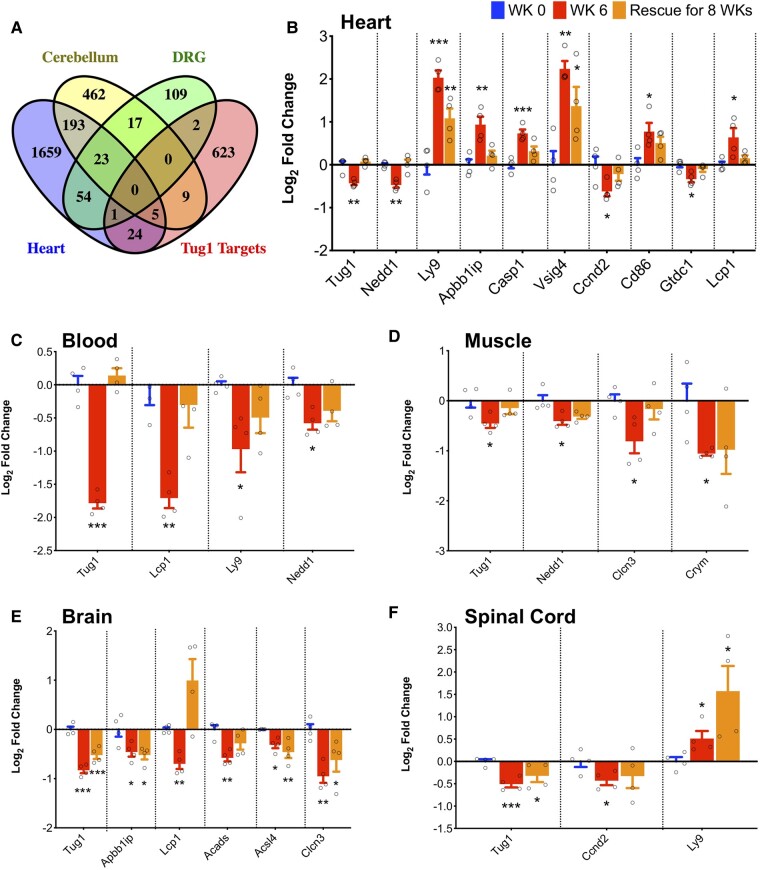
**Impact of *Tug1* downregulation on its target genes in various tissues of FRDAkd mice.** (**A**) Venn diagram illustrating the cross-comparison of *Tug1* targets identified from RNA pull-down assay using *Tug1*-transfected human glioma cells and other experimental data, with the list of differentially expressed genes uncovered via transcriptomic analysis in the heart, cerebellum and DRG of FRDAkd mice. (**B–F**) Expression changes in *Tug1* targets within (**B**) heart, (**C**) blood, (**D**) muscle, (**E**) brain and (**F**) spinal cord tissues of FRDAkd mice treated with dox for 0 and 6 weeks (*Fxn* knockdown), followed by an 8-week rescue phase (*Fxn* recovery). These alterations were assessed through qRT-PCR experiments. Four samples were taken at each time point (*n* = 4). One-way ANOVA was used for statistical analysis. Data are presented as mean ± SEM, with significance denoted as *P ≤ 0.05, **P ≤ 0.01 and ***P ≤ 0.001.

*Tug1* functions as a multifunctional regulatory factor and influences a range of cellular mechanisms that are disrupted in Friedreich's ataxia, including cell proliferation,^23-25^ apoptosis,^26-30^ cell cycle^24,26,31,32^ and mitochondrial bioenergetics.^33^ To further examine the role of *Tug1* in Friedreich's ataxia and validate its potential to serve as a biomarker, we investigated the downstream target genes of *Tug1*. One study identified 630 genes as *Tug1* targets using a modified RNA pull-down assay with promoter microarray analysis in BrU-labelled *Tug1*-transfected glioma cells.^36^ Cross-comparison of these 630 targets of *Tug1* combined with 33 experimentally verified *Tug1* targets against the list of differentially expressed genes in the heart, cerebellum and DRG of FRDAkd mice led to the identification of 40 overlapping genes (Fig. 3A). To validate this, we manually selected the top 16 *Tug1* targets based on differential expression *P*-value, expression in multiple tissues and association with cellular processes affected in Friedreich's ataxia as evidenced by the literature. The selected genes for validation were *Vsig4*, *Nedd1*, *Mmp2*, *Casp1*, *Lcp1*, *Acads*, *Bdnf*, *Acsl4*, *Cd86*, *Ly9*, *Gtdc1*, *Omg*, *Clcn3*, *Apbb1ip*, *Crym* and *Ccnd2*.

We conducted qRT-PCR experiments on blood, heart, muscle, brain and spinal cord tissues after *Fxn* knockdown and rescue in FRDAkd mice to validate *Tug1* targets. Out of 16 genes tested, 9 in the heart, 5 in the brain, 3 in the muscle, 3 in the blood and 2 in the spinal cord were differentially expressed. *Nedd1*, for instance, was significantly downregulated in the blood, heart and muscle in FRDAkd mice treated with dox for 6 weeks (Fig. 3). Depletion of NEDD1, a centrosome-localized protein, is linked to senescence induction in mouse embryonic fibroblasts,^37^ a phenomenon also observed in frataxin-deficient human neuroblastoma cells.^38^ CCND2, a protein responsible for regulating cell cycle, was regulated by lncRNA *TUG1* in bladder cancer, and the authors also reported that expression of *CCND2* positively correlated with *TUG1* expression.^39^  *Ccnd2* expression was downregulated in the spinal cord and heart. *Clcn3*, a gene thought to affect vesicle trafficking and exocytosis, was downregulated in the muscle and brain. Disrupted *Clcn3* leads to impairment of acidification of synaptic vesicles, which causes severe neurodegeneration.^40^ With frataxin knockdown and the corresponding *Tug1* deficiency, *Lcp1* was notably downregulated in the blood and brain but upregulated in the heart. *Ly9* exhibited downregulation in the blood and upregulation in the spinal cord and heart. Both *Lcp1* and *Ly9* have been implicated in T-cell activation,^41,42^ essential for immune system regulation. In FRDAkd mice, immune system activation was among the earliest pathways affected after frataxin knockdown.^13^ We also observed that several of these target genes including *NEDD1*, *CASP1*, *CD86*, *LY9*, *OMG*, *CCND2* and *CRYM* showed differential expression in Friedreich's ataxia patients compared with age- and sex-matched controls. This was evidenced in a data set comprising 183 human blood samples, which included 72 individuals with Friedreich's ataxia, 68 carriers and 43 unaffected controls (Supplementary Fig. 3). These findings strengthen the case for *Tug1* as a promising biomarker for Friedreich's ataxia, given the significant alterations in its downstream targets caused by *Fxn* knockdown in FRDAkd mice.

### RNA pull-down analysis of *Tug1* target gene associations in FRDAkd heart tissue

**Figure 4:**
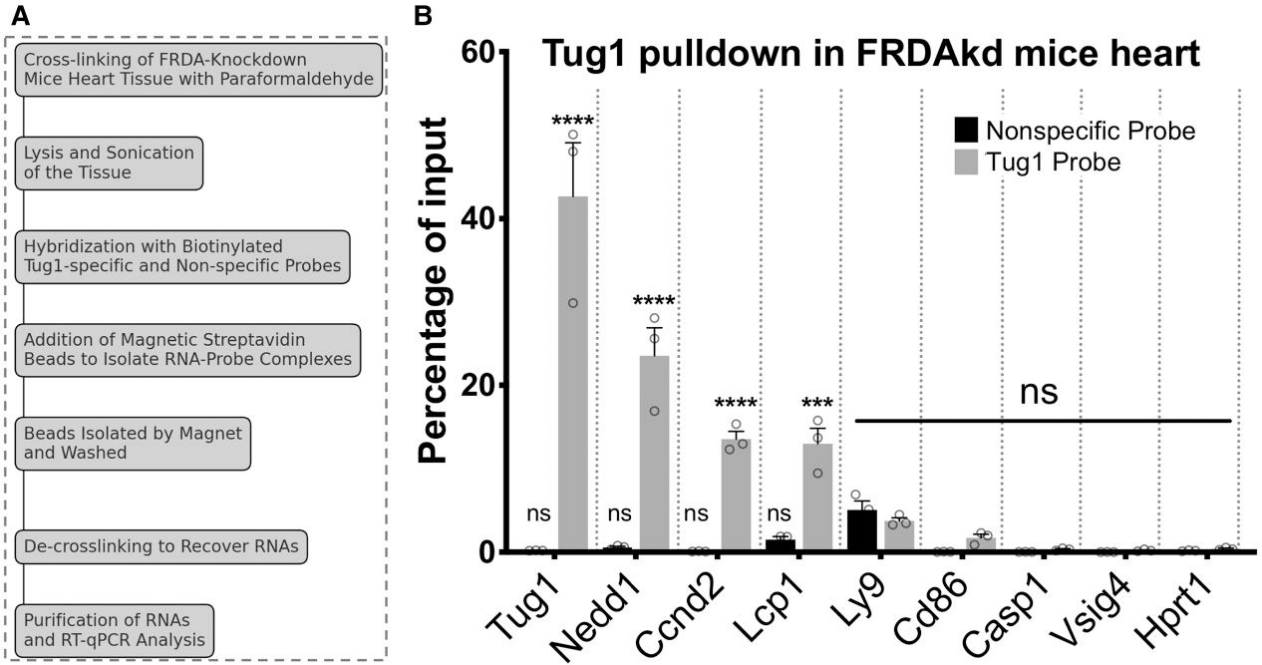
***Tug1* target enrichment in the heart tissue of FRDAkd mice revealed through RNA pull-down experiment.** (**A**) A schematic of the RNA pull-down experiment, where biotinylated probes were designed and used to isolate *Tug1* and its target genes using streptavidin beads. A non-specific probe served as a negative control. (**B**) Bar graph representing the percentage of input enrichment for several target genes, showing their significant association with *Tug1*. Each target gene is presented as a separate bar in the graph. These gene enrichments were determined by performing quantitative PCR on the *Tug1* RNA pull-down samples derived from the heart tissue of FRDAkd mice in the absences of frataxin knockdown (*n* = 3). A two-way ANOVA was used to evaluate statistical significance. Data are shown as mean ± SEM, with ***P ≤ 0.001 and ****P ≤ 0.0001 indicating levels of significance. ‘ns' denotes not significant.

To further elucidate the interaction between *Tug1* and its target genes in the heart tissue of FRDAkd mice (without frataxin knockdown), we conducted an RNA pull-down experiment. Utilizing a data-driven method with biotinylated probes, we aimed to isolate and specifically identify the target genes binding to *Tug1* (Fig. 4A). A subsequent analysis using quantitative PCR on the *Tug1* RNA pull-down samples obtained from the heart tissue of FRDAkd mice shed light on the enrichment of specific genes, reinforcing the interactions between *Tug1* and its target genes. Through this meticulous approach, we successfully validated the significant association of *Tug1* with several target genes, namely *Nedd1*, *Ccnd2* and *Lcp1* (Fig. 4B). These results not only suggest but also statistically associate *Tug1*'s involvement in key molecular mechanisms of Friedreich's ataxia, warranting further investigation into its functional roles and potential as a therapeutic target.

### Quantification of *TUG1* levels in Friedreich's ataxia patients and correlation analysis with key clinical parameters

**Figure 5:**
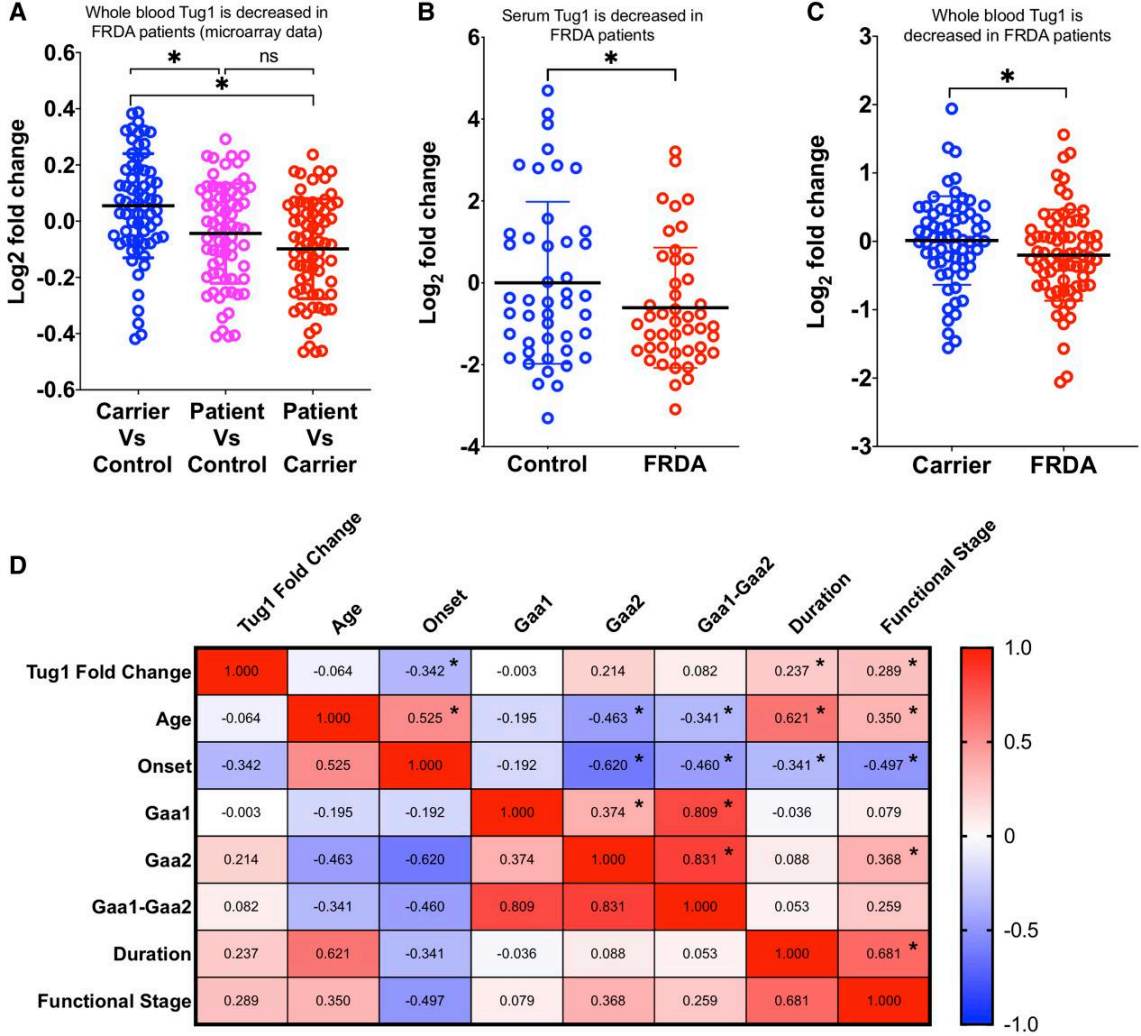
***TUG1* expression levels are reduced in Friedreich's ataxia patients.** (**A**) Scatter plot comparing whole-blood *TUG1* expression levels among age- and sex-matched Friedreich's ataxia patients (*n* = 72), heterozygous carriers (*n* = 68) and healthy controls (*n* = 43), using the GEO microarray data set GSE102008. Significance was assessed using one-way ANOVA with Holm–Sidak's multiple comparisons test. Data points are represented individually, with mean ± SD. *P < 0.05. ‘ns' denotes not significant. (**B**) Serum *TUG1* expression levels in Friedreich's ataxia patients (*n* = 45) exhibit significant downregulation compared with healthy controls (*n* = 45), as determined by RT-qPCR. Wilcoxon signed-rank test was performed. *P < 0.05. (**C**) Scatter plot illustrating the comparison of whole-blood *TUG1* expression levels between age- and sex-matched Friedreich's ataxia patients (*n* = 72) and heterozygous carriers (*n* = 66) via RT-qPCR. *P < 0.05 versus control group (Wilcoxon signed-rank test was performed). (**D**) Heat map displaying the correlation matrix between blood *TUG1* expression levels and various demographic characteristics of Friedreich's ataxia patients. GAA1 and GAA2 lengths represent the number of GAA triplet repeat expansions in the first and second alleles, respectively, of the FXN gene, which are characteristic of Friedreich's ataxia. Cell shading ranges from intense red indicating strong positive correlations to white indicating zero correlations to intense blue indicating strong negative correlations, reflecting the strength of associations. Pair-wise Pearson correlation coefficients are displayed in each cell, with stars marking significance at *P* < 0.05.

To investigate *TUG1* expression levels in patients with Friedreich's ataxia, we analysed the publicly available microarray data set GSE102008. Our comparative evaluation focused on whole-blood *TUG1* expression levels across age- and sex-matched individuals, including Friedreich's ataxia patients (*n* = 72), heterozygous carriers (*n* = 68) and healthy controls (*n* = 43). By employing a one-way ANOVA with Holm–Sidak's multiple comparisons test, we identified a significant downregulation of *TUG1* expression in the FRDA cohort (Fig. 5A). Given the previous detection of *TUG1* from human serum samples in patients with multiple myeloma,^43^ we explored its expression in Friedreich's ataxia serum samples. Our analysis of serum samples from 45 patient serum and 45 healthy control serum samples showed a significant downregulation of serum *TUG1* expression levels in Friedreich's ataxia patients compared with healthy controls, as confirmed through RT-qPCR, employing the Wilcoxon signed-rank test (Fig. 5B). We also examined and quantified the whole-blood *TUG1* expression levels between age- and sex-matched Friedreich's ataxia patients (*n* = 72) and heterozygous carriers (*n* = 66). Through RT-qPCR, we detected a significant downregulation (*P* < 0.05) in comparison with the control group (Wilcoxon signed-rank test; Fig. 5C). The results collectively demonstrated a marked downregulation of *TUG1* expression in both whole blood and serum from Friedreich's ataxia patients (Fig. 5A–C). Subsequently, to uncover the functional relevance of *TUG1* expression, we constructed a correlation heat map that revealed the relationship between blood *TUG1* expression levels (fold-change) and various demographic and clinical characteristics of Friedreich's ataxia patients. The correlation studies determined a substantial inverse correlation between *TUG1* fold-change and disease onset (Fig. 5D). Notably, we detected positive correlations with disease duration and the functional disability stage (FDS) score, which are clinical parameters known to directly influence each other (Fig. 5D). In summary, our thorough examination of *TUG1* expression levels across multiple cohorts and experimental frameworks has unveiled a distinct gene expression profile in Friedreich's ataxia patients. This novel understanding not only underscores the potential of *TUG1* as a therapeutic target but also lays the groundwork for subsequent investigations into the molecular pathways modulated by *TUG1* in Friedreich's ataxia.

### Regression analyses on *TUG1* expression levels and Friedreich's ataxia clinical variables

**Figure 6:**
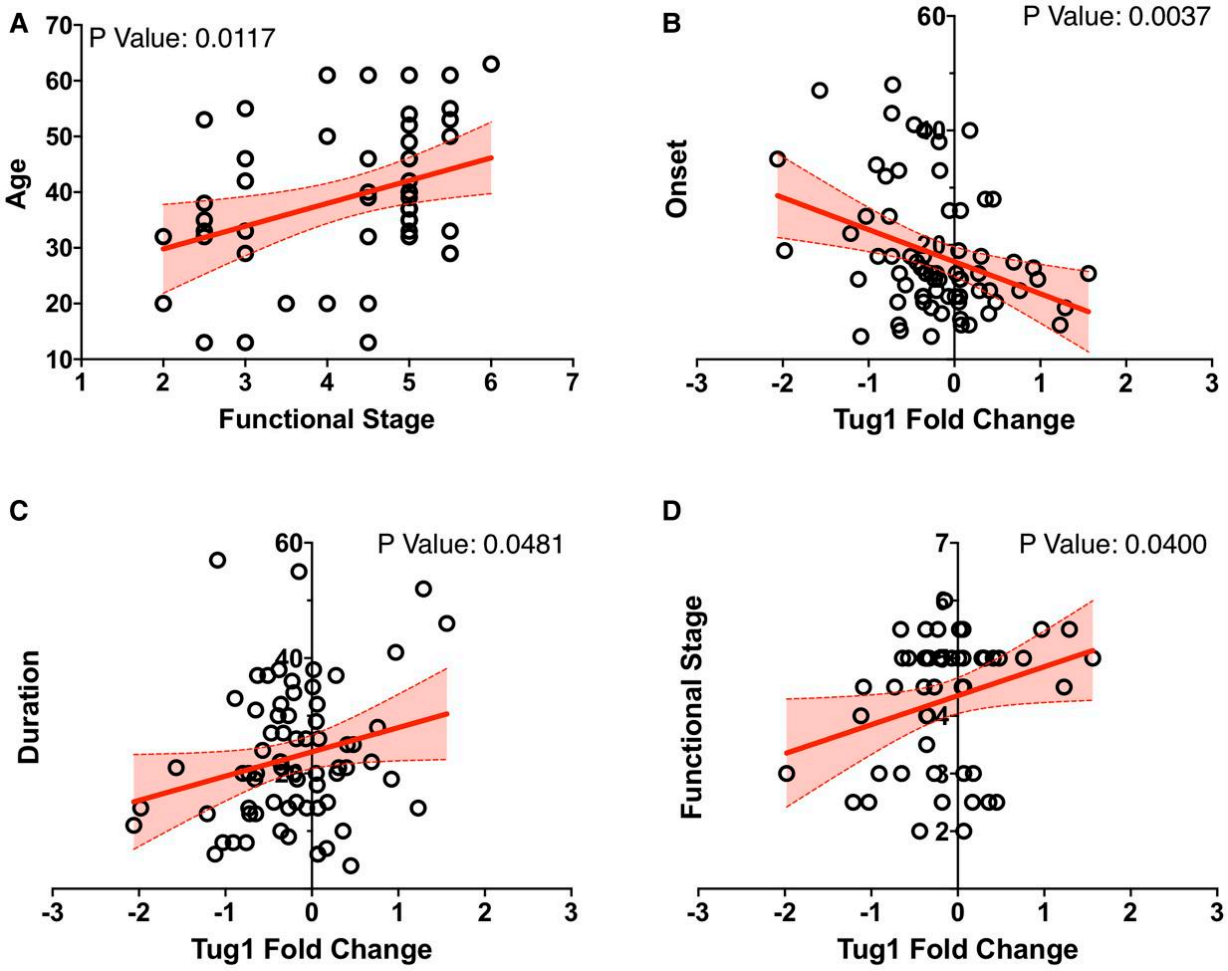
**Significant correlation between *TUG1* expression levels and clinical phenotypes in Friedreich's ataxia patients.** (**A–D**) Scatter plots and linear regression models illustrate the relationship between *TUG1* expression levels and various clinical phenotypes. (**A**) As anticipated, a significant association is observed between the FDS and age. (**B**) A significant negative correlation is present between disease onset and *TUG1* fold-change (*P* < 0.05). (**C**) A substantial positive correlation exists between *TUG1* fold-change and disease duration (*P* < 0.05). (**D**) The FDS also shows a significant positive correlation with *TUG1* fold-change, highlighting its potential as a biomarker for disease severity.

Given the evidence indicating significant correlations between *TUG1* fold-change and various factors including disease onset, duration and the FDS score in Friedreich's ataxia, we further explored these relationships through linear regression analyses. As expected, a key observation emerging from these analyses was a marked association between the FDS and age, emphasizing the clinical significance of these parameters in predicting the progression of the disease (Fig. 6A). Further, the linear regression results validated a negative correlation between *TUG1* fold-change and disease onset (*P* < 0.0037; Fig. 6B), reinforcing the relevance of *TUG1* in understanding disease onset. Additionally, positive correlations were established between *TUG1* fold-change and both disease duration (*P* < 0.04) and the FDS score (*P* < 0.04; Fig. 6C and D).

To further examine the impact of other variables, namely age, sex and GAA expansion (genetic marker of disease severity), on the correlation between *TUG1* fold-change, disease onset and FDS score, we conducted a multivariate regression analysis. The FDS emerged with an estimated coefficient of 0.1721, hinting at a positive relationship with *TUG1* fold-change. This infers that an increase in the FDS score is paralleled by a comparable increase in *TUG1* fold-change. However, this association did not reach the threshold of statistical significance (*P* = 0.0751), calling for further investigation. In contrast, the variable ‘disease onset’ was characterized by an estimated coefficient of −0.02661, representing a statistically significant negative relationship with *TUG1* fold-change (*P* = 0.0047). This compelling observation implies that elevated *TUG1* fold-change is associated with earlier disease onset and hence, more genetically severe cases. Residual analysis further validated our regression model, with residuals showing no clear patterns of heteroscedasticity or non-linearity. This supports the robustness of our findings and underscores the significant association between *TUG1* levels and disease onset (Supplementary Fig. 4). Taken together, our findings suggest that patients with increased levels of *TUG1* typically experience an earlier disease onset and present with higher FDS scores. On the other hand, patients with lower *TUG1* expression tend to have a delayed disease onset and exhibit less severe disease manifestations. These results underscore the potential of *TUG1* as a crucial biomarker in the prognosis and management of Friedreich's ataxia.

## Discussion

Friedreich's ataxia is a neurodegenerative disorder characterized by complex molecular mechanisms and limited therapeutic interventions.^44^ A significant challenge in the management of Friedreich's ataxia is the rapid monitoring of disease progression and the expedited evaluation of the efficacy of potential treatments.^8^ This challenge motivated our study to explore potential blood-based molecular biomarkers for Friedreich's ataxia. Upon re-examining a comprehensive data set comprising 733 individuals, we identified 293 genes showing differential expression in Friedreich's ataxia patients compared with controls. These genes predominantly function in immune system activities, aligning with existing literature that identifies immune activation as an early pathway regulated following frataxin (*Fxn*) knockdown.^11,12^ This finding not only lends credence to previous studies but also opens avenues for future research in identifying specific biomarkers for Friedreich's ataxia.

Extending the analysis to FRDAkd mice, we identified *Tug1* and *Slc40a1* as particularly promising biomarkers. These genes demonstrated consistent differential expression in both human patients and the FRDAkd mouse model. We validated the expression of *Tug1* and *Slc40a1* in both the mouse model and human blood samples, underscoring their potential as biomarkers and highlighting the benefits of minimally invasive sample collection. In FRDAkd mice, these genes were validated in different tissues primarily affected in Friedreich's ataxia, such as the heart, DRG neurons and the cerebellum, among others. Interestingly, *Tug1* and *Slc40a1* expression was significantly altered as early as 2 weeks following *Fxn* knockdown. This suggests the prospective value of these genes as early-stage biomarkers, whose expression is directly influenced by *Fxn* levels.

The FRDAkd mouse model was instrumental in assessing the correlation between frataxin levels and candidate biomarkers. Importantly, *Slc40a1* (ferroportin), involved in iron metabolism linked to Friedreich's ataxia pathology, showed tissue-specific expression changes after *Fxn* knockdown and partial restoration upon *Fxn* rescue. This observation correlates with previous findings that have implicated iron metabolism dysregulation in Friedreich's ataxia pathology,^45,46^ adding an extra layer of complexity and importance to our study. These collective findings contribute to a deeper understanding of the molecular mechanisms underpinning Friedreich's ataxia and underscore the potential utility of *Slc40a1* as a therapeutic marker.

lncRNAs such as *TUG1* have multifaceted roles in cellular biology, particularly in the epigenetic regulation of transcription.^47-49^ Our findings establish *Tug1* as a key molecular player that is downregulated in multiple tissues affected by Friedreich's ataxia pathology, specifically in response to *Fxn* knockdown. Given that *TUG1*’s involvement extends to cellular processes such as cell proliferation,^23-25^ apoptosis^26-30^ and mitochondrial bioenergetics^33^—processes that are also disrupted in Friedreich's ataxia—the importance of *TUG1* in Friedreich's ataxia pathogenesis becomes increasingly evident. We observed a stark downregulation of *Tug1* in various tissues, with an exception in the liver, upon *Fxn* knockdown. Its high expression and unchanged levels in the liver may imply tissue-specific functions of *Tug1*. Additionally, it is plausible that the half-life of *Tug1* RNA in the liver tissue is longer compared with other tissues. Strikingly, the levels of *Tug1* were partially restored following *Fxn* recovery, particularly in heart and muscle tissues. This emphasizes *Tug1*'s potential as a highly specific biomarker for Friedreich's ataxia.

Our analysis further extends to the exploration of *Tug1* and *Slc40a1* expression in whole blood across 11 time points during both *Fxn* depletion and recovery stages. Remarkably, *Tug1*’s strong linear correlation with *Fxn* levels, especially in comparison with *Slc40a1*, makes it a more suitable candidate as a peripheral biomarker. The trend of *Tug1* expression was statistically significant, showing a linear decrease during the *Fxn* depletion phase and a linear increase during the *Fxn* rescue phase. It is important to note that our findings rule out the confounding influence of dox treatment in FRDAkd mice, strengthening *Tug1*’s candidacy as a Friedreich's ataxia-specific biomarker. Our study also sheds light on the specific downregulation of *Tug1* in the cerebellum, which is crucial given the extensive cerebellar damage observed in Friedreich's ataxia.^32,33^ The early detection of *Tug1* downregulation in the cerebellum and its strong linear correlation with *Fxn* levels provide compelling evidence for *Tug1*’s importance in understanding the regional specificity of neuronal damage in Friedreich's ataxia.^50-53^ Ultimately, our work delves into the downstream target genes of *Tug1*, which include genes implicated in cellular processes disrupted in Friedreich's ataxia. For instance, *Nedd1*, a gene involved in cellular senescence,^37^ exhibited significant downregulation in multiple tissues in FRDAkd mice, reinforcing the vital role *Tug1* may play in the cellular biology disrupted in Friedreich's ataxia. Another notable gene, *Ccnd2*, which is implicated in cell cycle regulation,^54^ was also known to be regulated by *Tug1* and showed tissue-specific alterations. We observed varying expressions of *Tug1* targets across different tissues, indicative of *Tug1*'s multifaceted roles in Friedreich's ataxia. This suggests its potential influence on disease progression in a tissue-specific manner. Such variation aligns with current literature on the diverse functions of lncRNAs and their context-dependent regulatory mechanisms. These findings solidify the case for *Tug1* not just as a biomarker but as a critical molecular component in understanding the complex mechanisms underlying Friedreich's ataxia pathogenesis.

Our investigation into *TUG1* expression in Friedreich's ataxia patients, heterozygous carriers and healthy controls, utilizing public microarray data sets^14^ and RT-qPCR, reinforces *TUG1*'s potential as a biomarker. The results show a notable downregulation of *TUG1* in both whole blood and serum of Friedreich's ataxia patients. The linear and multivariate regression analyses highlight a significant association between *TUG1* levels and clinical variables such as disease onset, duration and the FDS score. Notably, we observed that elevated *TUG1* fold-change correlates with earlier disease onset, suggesting its utility in disease monitoring and therapeutic development. Furthermore, *TUG1* exhibits a complex relationship with both age of onset and disease duration in Friedreich's ataxia, indicating its diverse roles across different disease stages. Elevated levels of *TUG1* in the early stages may indicate early onset, while increases during disease progression could contribute to greater severity and longer duration. This intricate involvement of *TUG1* in Friedreich's ataxia's neurodegenerative pathology underscores its multifaceted role, emphasizing the need for further exploration of its potential as a biomarker across various stages of the disease. Periodic assessments of *TUG1* levels, using easily accessible samples such as blood and serum, could serve as tools for tracking disease progression and severity. The implications for therapeutic development are profound, potentially transforming drug development processes and guiding targeted interventions. Insights into the molecular pathways of *TUG1* could lead to targeted therapies, transforming Friedreich's ataxia drug development.

Our study employs a comprehensive approach and diverse sample analysis, incorporating both human and mouse model data, to substantiate the validity of our findings. The use of robust validation methods and the FRDAkd mouse model^13^ further enhances the reliability of our study, positioning non-coding RNA *TUG1* as a promising biomarker for Friedreich's ataxia. Nevertheless, our study has limitations that warrant attention. Despite our efforts to eliminate confounding effects, the potential impact of unrecognized confounding variables on our results cannot be completely ruled out. Therefore, clinical validation through trials is essential for further substantiation. Additionally, our findings require replication in larger cohorts to establish broader validity, and longitudinal studies are crucial for understanding the long-term association of *TUG1* with disease progression. Furthermore, the complex interplay between *FXN*, *TUG1* and other cellular processes necessitates more detailed investigation.

In conclusion, our rigorous study underscores *TUG1*'s critical role as a prospective blood-based biomarker for Friedreich's ataxia. Utilizing a robust methodology and in-depth analyses, we have confirmed the functional importance of *Tug1* and its downstream targets in tissues specific to Friedreich's ataxia. Importantly, our data reveals a strong correlation between *TUG1* expression and key clinical indicators, such as disease onset and FDS scores, further establishing its clinical significance. *TUG1* stands out as an early-stage, blood-based marker, thereby emphasizing its potential for minimally invasive diagnostic applications with substantial clinical and therapeutic implications. As such, *TUG1* offers a promising path for both monitoring the disease and guiding therapeutic development, contributing to improved patient care and deeper understanding of this complex neurodegenerative disorder. Future research should aim to validate these findings through larger and more diverse patient studies while also focusing on the translational potential of these scientific insights into effective clinical applications.

## Supplementary Material

fcae170_Supplementary_Data

## Data Availability

The raw gene expression data for Friedreich's ataxia patients can be accessed via the NCBI Gene Expression Omnibus (GEO) database, under the accession number GSE102008. Similarly, gene expression data sets for FRDAkd mice are publicly available on the GEO database under the accession number GSE98790. All additional data pertinent to this study can be obtained upon reasonable request directed to the corresponding author.
