# Supplementary material for: Long non-coding RNA TUG1 is downregulated in Friedreich’s ataxia
Source: Brain Commun. 2024 May 15;6(3):fcae170. doi: 10.1093/braincomms/fcae170 (PMC11154142; doi:10.1093/braincomms/fcae170)
Supplement: fcae170_Supplementary_Data [file fcae170_supplementary_data.pdf]

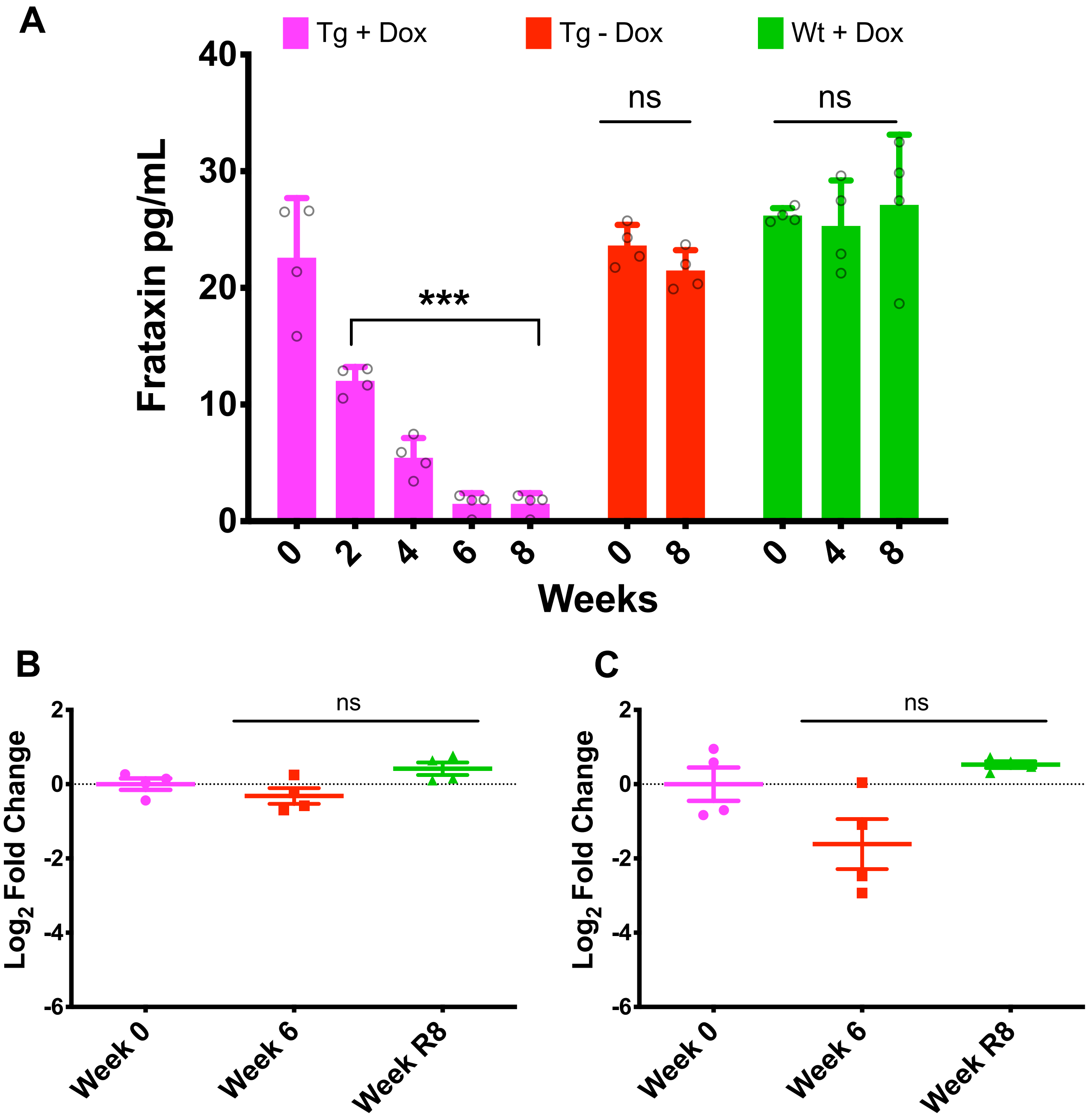

Supplementary Figure 1. Assessment of FXN Levels in FRDA-Knockdown Mice and Gene Expression Levels in Wildtype Mice. (**A**) Relative quantification of FXN levels in heart tissue samples from FRDA-knockdown mice. Three different groups were tested: Transgenic with doxycycline treatment (Tg + Dox), transgenic without doxycycline treatment (Tg - Dox), and wildtype with doxycycline treatment (Wt + Dox). The FXN levels were measured via ELISA assay. Each group consisted of four samples (n = 4). (**B-C**) Expression levels of (**B**) *Tug1* and (**C**) *Slc40a1* in wildtype mice treated with doxycycline, followed by a recovery (R) phase. One-way ANOVA and Welch's t-test were utilized for statistical analyses. Data are presented as mean ± SEM, with *** denoting $p \leq 0.001$.

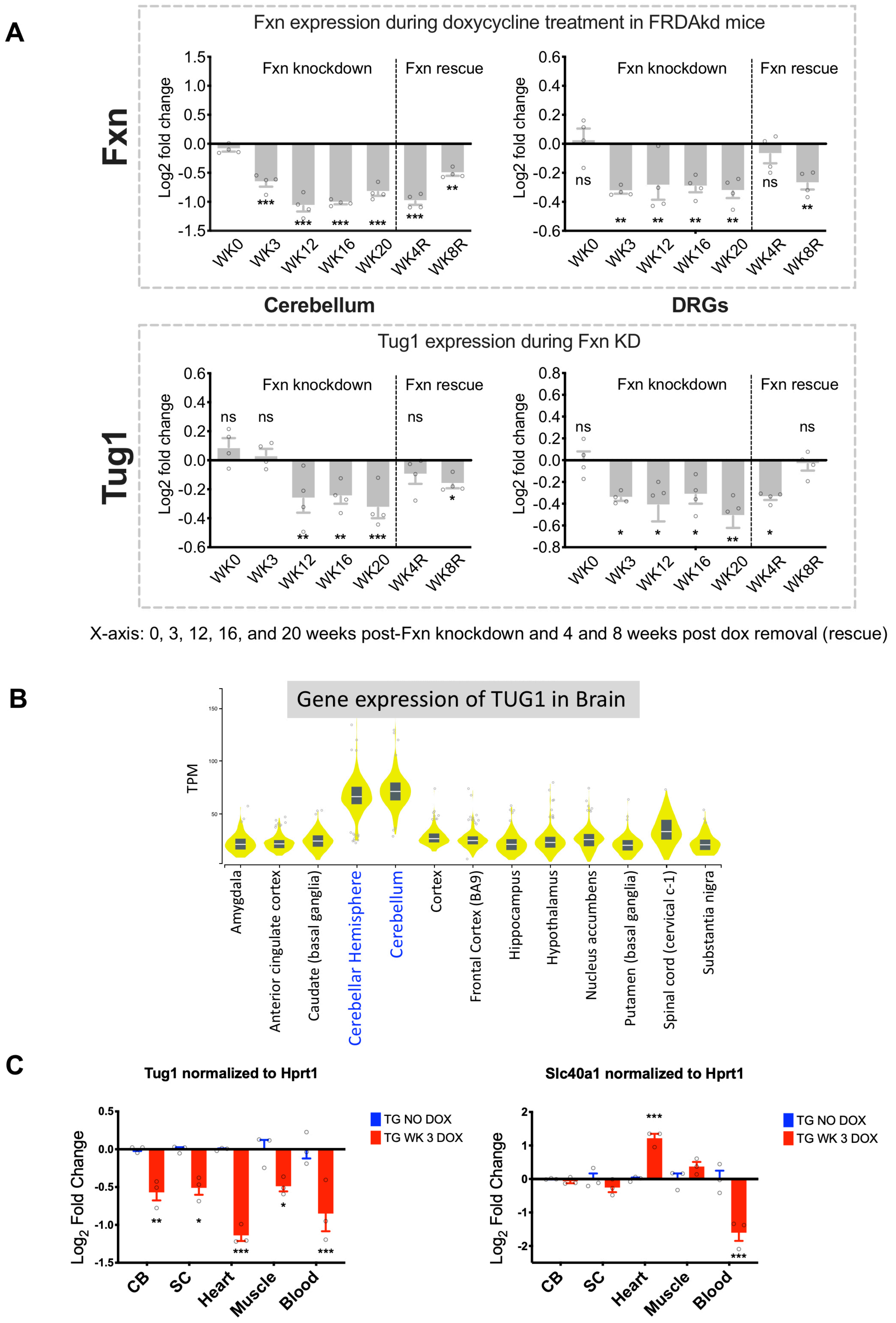
A
Fxn expression during doxycycline treatment in FRDAkd mice
Fxn
Log2 fold change
Fxn knockdown
Fxn rescue
ns
WK0
WK3
WK12
WK16
WK20
WK4R
WK8R
Cerebellum
DRGs
Tug1 expression during Fxn KD
Tug1
X-axis: 0, 3, 12, 16, and 20 weeks post-Fxn knockdown and 4 and 8 weeks post dox removal (rescue)
B
Gene expression of TUG1 in Brain
TPM
Amygdala
Anterior cingulate cortex
Caudate (basal ganglia)
Cerebellar Hemisphere
Cerebellum
Cortex
Frontal Cortex (BA9)
Hippocampus
Hypothalamus
Nucleus accumbens
Putamen (basal ganglia)
Spinal cord (cervical c-1)
Substantia nigra
C
Tug1 normalized to Hprt1
Slc40a1 normalized to Hprt1
Log2 Fold Change
TG NO DOX
TG WK 3 DOX
CB
SC
Heart
Muscle
Blood

Supplementary Figure 2. Correlation of *Tug1* Expression with *Fxn* Levels in FRDA-Knockdown Mice and *Tug1* Expression across Human Brain Regions. (**A**) Longitudinal analysis of *Fxn* and *Tug1* gene expression in the cerebellum and dorsal root ganglia (DRGs) of FRDA-knockdown mice (FRDAkd) during frataxin knockdown (at weeks 0, 3, 12, 16, and 20) and recovery (at weeks 4 and 8 – WK4R and WK8R). Four biological replicates were assessed for each time point. Statistical analysis was carried out using one-way ANOVA with post hoc multiple comparison test. (**B**) Violin plot illustrates the gene expression levels of TUG1 in various human brain regions, with data obtained from the GTEx database. There is notable emphasis on the expression levels of TUG1 in the cerebellum and cerebellar hemispheres, which are regions critically affected in FRDA. The expression values are normalized to Tags Per Million (TPM), as indicated on the y-axis. The brain regions examined are listed on the x-axis, with sample sizes ranging from 3 to 14 (n= 3-14). The individual circles represent actual data points, corresponding to the TUG1 expression level in each specific brain region. The central line within each box denotes the median value of TUG1 expression. The box itself represents the interquartile range (IQR) of the data, while the outer shape of the violin displays the kernel density estimation, indicating the distribution of the data. (**C**) Expression levels of *Tug1* and *Slc40a1* in cerebellum, spinal cord, heart, muscle, and blood samples from FRDA-knockdown mice, treated with doxycycline for three weeks, normalized to *Hprt1*. TG NO DOX = control transgenic animals without doxycycline treatment (blue), TG WK 3 DOX = transgenic animals with doxycycline treatment for three weeks (red). Three biological replicates (n = 3) were analyzed. Statistical analysis was carried out using two-way ANOVA with post hoc multiple comparison test. Data are presented as mean ± SEM; asterisks denote significance levels as * = $p \leq 0.05$, ** = $p \leq 0.01$, *** = $p \leq 0.001$.

TUG1

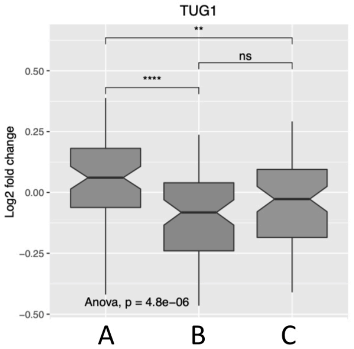

CASP1

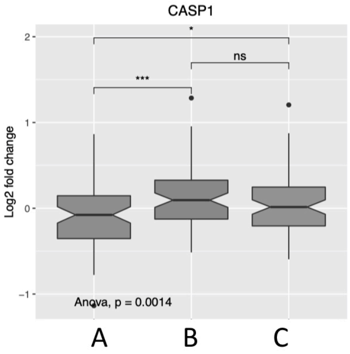

LCP1

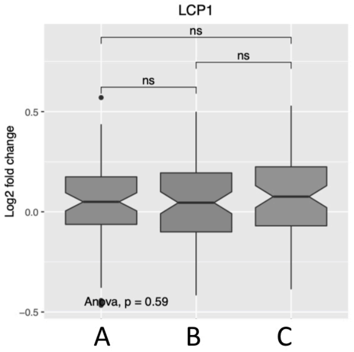

BDNF

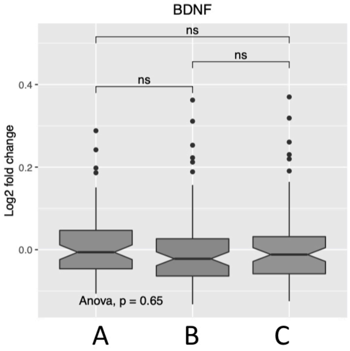

CD86

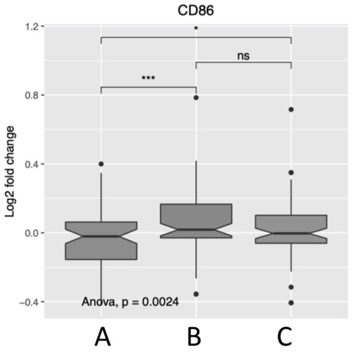

LY9

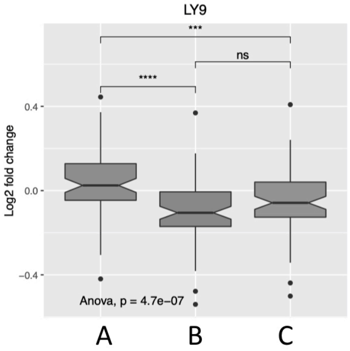

OMG

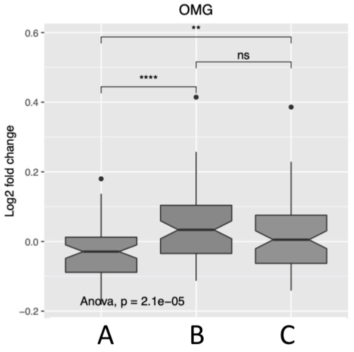

CCND2

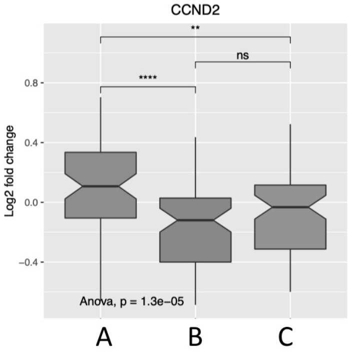

CRYM

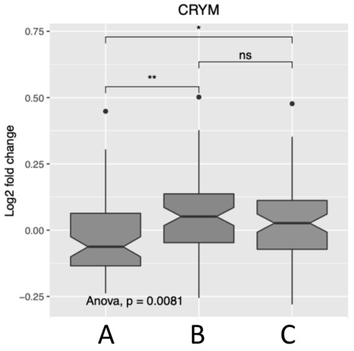

A = Carrier_Vs_Control
B = Patient_Vs_Carrier
C = Patient_Vs_Control

Supplementary Figure 3: Differential Expression of *TUG1* and Related Target Genes in FRDA. This figure illustrates boxplots of the expression levels for *TUG1* and eight other target genes (*CASP1*, *LCP1*, *BDNF*, *CD86*, *LY9*, *OMG*, *CCND2*, and *CRYM*) in whole blood samples from age- and sex-matched FRDA patients (n=72), heterozygous carriers (n=68), and healthy controls (n=43), as per the GEO microarray dataset GSE102008. The first column represents the comparison between carriers and controls (A), followed by comparisons of patients versus carriers (B) and patients versus controls (C). The median is denoted by the central line in each box, while the limits of the boxes indicate the upper and lower quartiles. Whiskers extend up to 1.5 times the interquartile range from the box. The Wilcoxon test assessed differences in expression between groups, revealing significant differential expression in FRDA patients compared to carriers and controls across multiple genes, with p-values indicated above each comparison. Values are presented as log2 fold-change with mean ± standard deviation (SD). Statistically significant differences are marked with asterisks (one-way ANOVA with Holm-Sidak's multiple comparisons test; asterisks denote significance levels as * = $p \leq 0.05$, ** = $p \leq 0.01$, *** = $p \leq 0.001$).

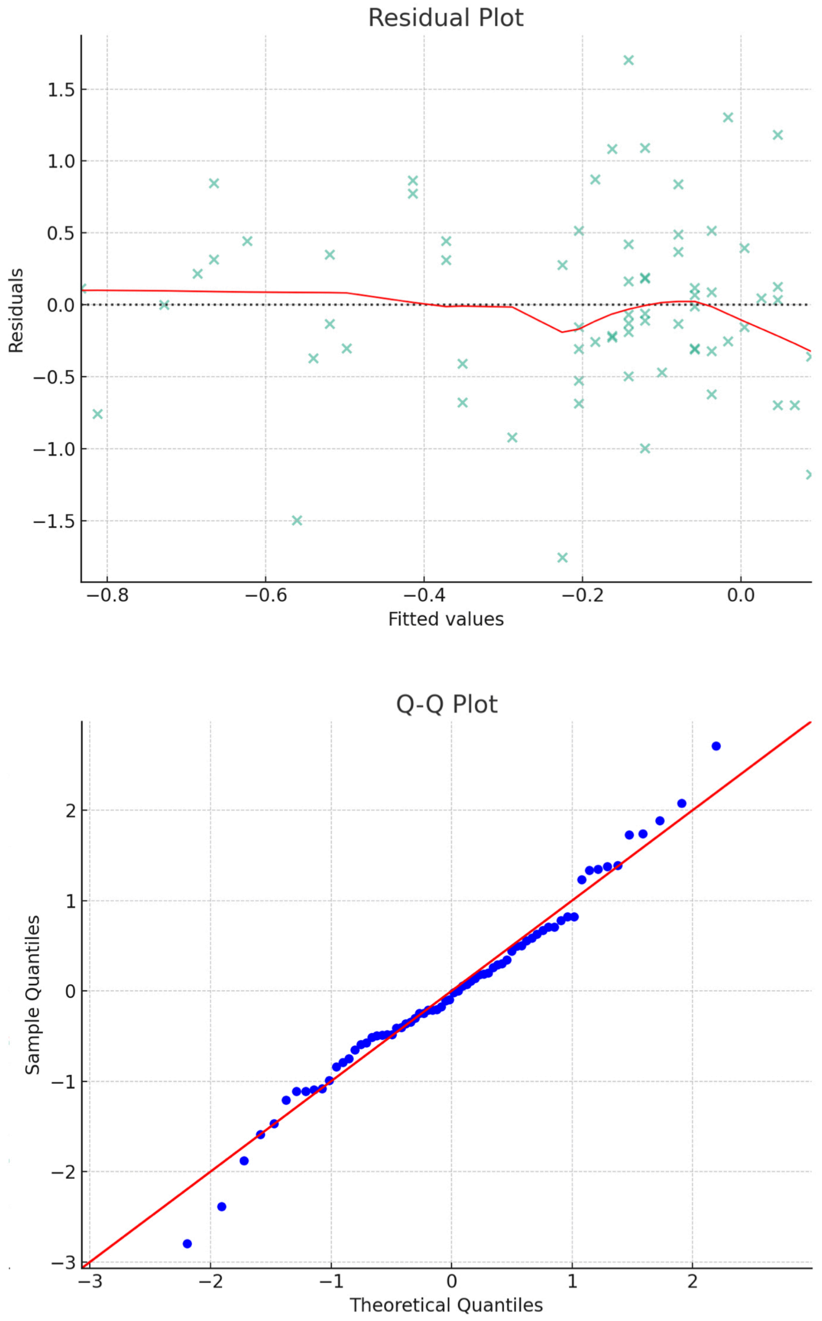

Supplementary Figure 4: Residual and Normal Q-Q Analysis of Regression Model with *TUG1* Levels as the Dependent Variable and Disease Onset as the Independent Variable. The top panel presents a Residual plot, where each data point corresponds to a specific observation plotted against its predicted value. The red Lowess line, ideally lying horizontally at zero if the model assumptions are upheld, exhibits minor deviation, demonstrating an acceptable model fit with no distinct residual patterns. The bottom panel exhibits a Normal Q-Q plot, which juxtaposes the distribution of residuals against the standard normal distribution. The scatter points signify residuals, largely aligning along the diagonal red reference line, thereby implying a predominantly normal distribution of residuals.
